# Seroprevalence of Gestational and Neonatal Toxoplasmosis as well as Risk Factors in Yaoundé, Cameroon

**DOI:** 10.1155/2022/6406259

**Published:** 2022-03-21

**Authors:** Joy Nkain Ayeah, Adesina Oladokun, Irene Ule Ngole Sumbele, Abiodun Olatunbosun Ilesanmi, Obase Ngemani Bekindaka

**Affiliations:** ^1^Department of Obstetrics and Gynaecology, University of Ibadan, Ibadan, Nigeria; ^2^Department of Zoology and Animal Physiology, University of Buea, Buea, Cameroon; ^3^Department of Microbiology and Immunology, Cornell College of Veterinary Medicine, Ithaca, New York, USA

## Abstract

**Background:**

Toxoplasmosis, caused by *Toxoplasma gondii* in pregnant women, is a significant public health problem due to risk of mother to child transmission. The aim of the study was to determine the seroprevalence of toxoplasmosis in pregnant women and corresponding cord blood among women attending Biyem-Assi and CASS Nkoldongo hospitals in Yaoundé, Cameroon.

**Methods:**

An institutional based cross-sectional study was conducted between June 2019 and May 2020 on 300 pregnant women from late second trimester to third trimester. A total of 259 cord blood samples were collected at birth from these women. *Toxoplasma gondii*-specific IgG and IgM antibodies in maternal and cord blood were detected using the Toxoplasma Enzyme Immunosorbent Assay kit, and potential risk factors captured through questionnaire were identified using binary logistic regression model. Statistical significance was measured at *P* < 0.05.

**Results:**

The overall seroprevalence of gestational and neonatal toxoplasmosis was 80% and 88%, respectively. IgG seropositivity was 72.7%, IgM only was 1.3% and cooccurrence of IgG/IgM was 6% amongst pregnant women. Out of 259 newborn cord bloods, 72.2% were positive for IgG only, 8.9% for IgM only, and 23.9% for both IgG/IgM. Pregnant women 15-24 years (AOR = 4.6, *P* = 0.011) and women with primary level of education (AOR = 3.9, *P* = 0.042) were significantly at risk of infection with *Toxoplasma gondii*.

**Conclusion:**

Gestational and neonatal toxoplasmosis appears to be more common with higher risk of infection in younger women and less educated women. Hence, these findings will serve as baseline data for further investigations on mother to child transmission of toxoplasmosis in Yaoundé and the need for reinforcement of pregnant women toxoplasmosis-related health measures.

## 1. Introduction

Toxoplasmosis is a zoonotic parasitic disease caused by *Toxoplasma gondii (T. gondii)*. Its definitive hosts are members of the cat family while its intermediate hosts include wide variety of animals and humans [1, 2]. *T. gondii* circulates in three infectious forms: the tachyzoite, the bradyzoite (able to form tissue cysts), and the sporozoites, formed within the oocysts [1]. *T. gondii* is ranked fourth among 24 most significant foodborne parasites by the World Health Organisation (WHO) and the United Nations Food and Agriculture Organization (FAO) [3]. Infection occurs through ingestion of oocysts in water or food or soil contaminated with cat faeces, raw or undercooked meat containing tissue cysts, and through transplacental transmission [4, 5]. Toxoplasmosis estimates show that one-third of the world's population is affected by latent toxoplasmosis and majority of infections are reported in South America and Africa [6]. Infections are most often self-limiting and asymptomatic in immune competent persons; however, infection could result in serious consequences in immune compromised persons [7]. In HIV/AIDS persons, toxoplasmosis is an important opportunistic infection and usually occurs as a result of reactivation of latent infection often manifesting as toxoplasmic encephalitis [8].

Gestational toxoplasmosis is an important public health problem due to the risk of mother to child transmission which may result in devastating consequences such as abortion, stillbirth, premature births, birth defects, congenital toxoplasmosis (CT) and other pregnancy outcomes [9]. More than 80% of pregnant women who acquire *T. gondii* infection remain asymptomatic; nonetheless, transplacental transmission to foetus remains possible and may be associated with devasting consequences [10]. The risk of mother to child transmission increases with increase in gestational age. For untreated pregnant women the transmission rate varies from 25% in the first trimester to 65% in the third trimester [11]. Among newborns, more than 60% cases of congenital toxoplasmosis are usually asymptomatic [11]. Since congenital transmission of *T. gondii* occurs mostly in women who acquire infection during gestation, determining whether *Toxoplasma* infection has occurred during pregnancy is critical [12]. Diagnosis involves detection of specific immunoglobulins (Ig) G and M antibodies by serology whereby IgM is a marker of acute or recent infection while IgG is a marker of past or chronic infection [13]. In addition, confirmatory tests such as the IgG avidity or PCR test is always useful in eliminating false positive IgM because its erroneous interpretation could be misleading [5]. Many studies indicate that gestational toxoplasmosis varies geographically. Existing reports showed a prevalence of 68.4% in Brazil [14], 6.4% in South Africa [15], 42.5% in Malaysia [16], 36.7% in France [17], 92.5% in Ghana [18], 5.87% in Zambia [19], 30.9% in Tanzania [20], and 20.3% in Burkina Faso [21]. In different regions of Cameroon, seroprevalence of over 50% have been reported [22–25], while other regions have had seroprevalence rates of less than 50% [26, 27].

Since majority of infections with *T. gondii* are asymptomatic, early detection, treatment, and primary prevention remains the best way to limit the risk of congenital infection [10]. A number of risk factors have been found associated with variations in seroprevalence of *T. gondii*, age, [28, 29] keeping pet cats, contact with soil, parity [30], and eating unwashed raw vegetables [10]. Nonetheless, conflicting results about which factors influence the vulnerability of humans to *T. gondii* infection have been reported. For instance, a study in Ethiopia [31] could not relate the high prevalence of *T. gondii* antibodies in pregnant women to any known risk factor. For proper implementation of primary preventive ways for effectiveness, knowledge of possible risk factors in each population needs to be frequently assessed to identify the risk factors specific to that population.

Instituted systematic screening of pregnant women throughout the gestational period in Europe has proven effective in detecting acute infections and congenital toxoplasmosis [11, 32]. Nevertheless, Cameroon does not have an instituted program for systematic screening of *T. gondii* infections in pregnant women or in newborns. Screening of mother and newborn at birth may be an effective strategy for identifying cases of congenital toxoplasmosis thus enabling immediate treatment and limiting its complications such as chorioretinitis later in life [33]. Despite the documented risk of mother to child transmission of *T. gondii* in literature, there is no comprehensive and documented survey on newborn cord samples for the case of Cameroon. The objective of the study was to determine the seroprevalence of gestational and neonatal toxoplasmosis as well as associated risk factors.

## 2. Methods

### 2.1. Study Design, Site, Population, and Ethical Aspects

This cross-sectional study was conducted in the Central Region of Cameroon, specifically in the city of Yaoundé. Yaoundé is the administrative and political capital of Cameroon, located between latitudes 3°47-3°56 North and longitudes 11°10-11°45 East at an altitude of 750m [34]. Yaoundé, a highly cosmopolitan city, has a population of over 2.6 million inhabitants regrouped from various ethnic groups in Cameroon. Yaoundé has a tropical climate of four seasons: 2 rainy seasons and 2 dry seasons. It has an average annual rainfall of 1643 mm for an average temperature of 23.7°C.

Two representative hospitals were selected from two district health areas: Biyem-Assi District Hospital and Centre d'Animation Sociale et Sanitaire (CASS) de Nkoldongo. These health institutions were selected due to their strategic locations that make them receive patients and pregnant women from all over the city. The study population comprised of pregnant women from 2^nd^ trimester to their 3^rd^ trimester or at term, between the ages of 15 and 49 years old living within or around the city of Yaoundé. Cord blood (CB) was collected after childbirth from each corresponding live born at birth. Participants were excluded from the study if they refused to sign the consent form, had incomplete data in the questionnaire, and/or had inadequate samples. This study was approved by the Institutional Review Board of the University College Hospital of Ibadan, Nigeria (Ref#18/0602) and the Cameroon National ethics Committee (No. 2019/11/55/CE/CNERSH/SP).

### 2.2. Sample Size and Sampling Procedures

Following Cochran's formula (1963) [35], a total sample size of 342 was calculated from a previous *Toxoplasma* seroprevalence of 65.5% among pregnant women in Douala, Littoral Region of Cameroon [22]. Sampling of pregnant women was done by convenience, and 360 pregnant women were approached with consent forms to sign after study was explained to them, but 310 fully consented to the study. However, only 300 pregnant women completed questionnaires and gave venous blood samples. This corresponds to a statistical power > 85% of the required sample size indicating that results obtained in this study are reasonably true. At delivery, only 259 cord blood samples were collected. Some women had complications and were transferred to referral hospital for delivery, while other deliveries were missed and 4 deliveries were stillbirth.

### 2.3. Sample Collection and Processing

Approximately 3-5 ml of venous blood from mothers and cord blood from newborn umbilical cord were collected into well-labelled sterile dry tubes each. The samples were placed on ice blocks and transported to the Immunology Laboratory at the Biotechnology Centre of Nkolbisson, Yaoundé. Samples from the mother were collected during pregnancy at late second trimester, third trimester, and even near delivery. Serum was obtained after centrifugation of blood samples was done at 2000 rpm for 10 minutes. The sera were preserved at -20°C until laboratory analysis.

### 2.4. Questionnaire Administration

Questionnaire was pretested on 10 pregnant women. Responses were evaluated and questions adjusted for clearer understanding. Questionnaires were formulated based on literature and administered in English or French depending on language preference of participants. Information on sociodemographic factors (age, marital status, educational status, profession, and household income), obstetrical status (antenatal (ANC) visits, gravidity, parity, history of abortion, and history of stillbirth), nutritional habits (tasting of meat undercooked meat, consumption of grilled meat, consumption of bushmeat, eating unwashed vegetables/fruits, and drinking water source), farm work, cats at home, cats in neighbourhood, and HIV status was collected.

### 2.5. Serological Method

The enzyme-linked immunosorbent assay to detect specific IgG and IgM against toxoplasmosis was carried out using commercial anti *Toxoplasma* immunoassay kits, Rapid Labs limited (Toxo EIA Rapid Labs kit, UK, Ltd.). The analysis was conducted following the manufacturer's instruction with a little modification. Results were read using the ELISA microplate reader, and index values less than 1.1 and greater than 1.1 were considered negative and positive, respectively, for both antibodies. The same serological kit (same manufacturer) was used for both mother and cord blood samples in order to preserve the internal validity of this study.

### 2.6. Statistical Analysis

Data was subjected to statistical analysis in IBM-SPSS™ (Statistical Package for the Social Sciences version 21, SPSS Inc., Chicago, IL, USA). Descriptive statistics were presented as frequencies, means, and percentages. Chi-square (*χ*^2^) was used to measure associations between *Toxoplasma* infection (positive and negative) and characteristics of pregnant women through cross tabulations. Data from women with known IgG and IgM serostatus were included in the analysis. Binary logistic regression analysis for *Toxoplasma* infection (positive or negative) was performed to examine the level of associations. Only variables that had a threshold of *P* ≤ 0.20 were introduced into the binary logistic model. Binary logistic regression was conducted for specific IgG serostatus only since IgM anti-*T*. *gondii*-positive women were few. Crude odd ratios (COR) and adjusted odd ratios (AOR) were obtained. *P* value < 0.05 was considered statistically significant.

## 3. Results

### 3.1. Description of Study Characteristics of Pregnant Women

Pregnant women included in the study ranged between the ages of 16 and 41 with a mean age of 28.05 ± 5.83 years. Most of the women (55.3%, 166/300) in the study were aged 24 to 34 years. Majority of women (72%) had a secondary education and 62% of women had an income between 50,000 and 100,000FCFA (Table 1). With respect to profession, students, self-employed, and unemployed persons had similar proportions (24.7%, 26.7%, and 27%, respectively). A little fraction of the population, 27(9%) were HIV positive. Majority of the participants (52.3%, 157) already had multiple pregnancies and had 2-4 children (53.7%). Only 15 (5%) had a history of stillbirth while 97 (32.3%) had a history of spontaneous abortion. Most of the women consumed bush meat (58.7%, 176) and grilled meat locally called “soya” (88%, 264). A minority of pregnant women (9.7%, 29) reported that they drank water from untreated water or mixed water sources where treated water (tap water, spring water, and mineral water) and untreated/mixed sources (streams alone or streams and treated water). Only 19.7% of subjects were involved in farm work and 22% of the pregnant women had cats at home while 33.2% had cats and/or other animals in the neighbourhood as shown in Table 1. On the other hand, a total of 259 cord blood samples were collected from live births.

### 3.2. Seroprevalence of *T*. *gondii* in Pregnant Women and Corresponding Newborns

Of the 300 women tested for specific IgG and IgM antibodies against gestational toxoplasmosis, a total of 242 (80%) were positive. Seropositivity for *Toxoplasma* IgG only was highest (72.7%, 218) classified as latent or chronic infection. Seropositivity for *Toxoplasma* IgM only was 1.3% (4), and the cooccurrence of IgG/IgM only was 6% (18) classified as acute infections. Thus, the total occurrence of specific IgMs were 7.3%. While those who tested negative for both *Toxoplasma* antibodies (IgG, IgM) were classified as susceptible. The age-specific seropositivity alongside other sociodemographic and clinical factors is displayed in Table 2. There was an incremental seroprevalence with age that approached significance (*P* = 0.051). The seroprevalence of specific antibody IgG and IgM in association with other factors were not statistically significant as shown in Table 2.

Among the 259 umbilical cord blood samples tested for specific IgG and IgM antibodies against neonatal toxoplasmosis, a total seroprevalence of 88% (228) was obtained. Cord blood (55.2%, 143) was seropositive for specific IgG only, 8.9% [23] seropositive for IgM only, and 23.9% (62) seropositive for both IgG/IgM. An intriguing observation of 41.5% (34/82) newborn CB positive for IgM but born from totally negative mothers was noted.

### 3.3. Risk Factors Associated with *T. gondii* among Pregnant Women

Following a selection criterion (*P* ≤ 0.20) of independent variables in the univariate analysis for entry into the binary logistic regression model, only age, educational status, household income, parity, history of stillbirth, presence of cats at home, and presence of cats in neighbourhoods were included in the model. As shown in Table 3, age and educational status were identified as significant risk factors of infection. Pregnant women 15-24 years old had 4.6-fold odds of being infected with *T. gondii* (AOR = 4.649, *P* = 0.011). On the other hand, women with primary educational level were 3.9 times more likely to be infected with *T. gondii* (AOR = 3.940, *P* = 0.042) when compared with their counterparts. While women who had a history of stillbirth were significantly protected against *T. gondii* IgG seropositivity (AOR = 0.206, *P* = 0.02), other factors like household income, parity, presence of cats at home, or cats/animals in the neighbourhood were not significantly associated with *T. gondii* IgG seroprevalence (Table 3).

## 4. Discussion

Gestational toxoplasmosis is endemic and a disease of public health significance in Cameroon. Unlike many studies, this study concurrently determined the seroprevalence of toxoplasmosis in pregnant women and newborn cord blood. The study showed that the seroprevalence of toxoplasmosis in pregnancy was 80%. This is similar to 80.3% obtained in the Democratic Republic of Congo (DRC) amongst pregnant women [36]. However, this was lower than 85.5% reported in Ethiopia [37] and 92.5% in Ghana [18]. In Cameroon, the seroprevalence was similar to a recent (78.6%) study in Douala [22] and an earlier study in Yaoundé (77.1%). [25] On the other hand, the seroprevalence obtained in this study is substantially higher than most studies within Cameroon: 54.5% in Njinikom, North West Region, [24] 70% in Yaoundé [23], 32.5% in Buea [27], and 45.5% in Mbouo-Bandjoun, West Region [26]. Such variations in the seroprevalence of toxoplasmosis may be due to differences in geographical locations, characteristics of the pregnant women such as age, educational level, handling of cats, hygiene, and feeding-related practices of toxoplasmosis. [38] The use of different serological methods and the difference in sensitivity may also be responsible for the divergences. [39]

Majority (72.7%) of pregnant women were reactive for *Toxoplasma* IgG antibody only, indicating that most infections were past or latent infections. This is a common trend across most studies where majority of infections are often past infections with few or no cases of *Toxoplasma*-specific IgM, the marker of recent infection. Its presence in the absence of IgG evokes a recent infection and needs confirmatory test. [40] In this study, those that were exclusively positive for specific *Toxoplasma* IgM in pregnant women (1.3%) was similar to that reported in the local studies [23, 24] in Cameroon. During a *Toxoplasma* infection, IgM antibodies tend to appear earlier and so are the first class of antibodies detected after a primary infection. However, they also decline very rapidly than IgG antibodies and this is probably why most *Toxoplasma* infections detected are IgG [12]. This could also be an indication of low active transmission of toxoplasmosis. The high seroprevalence of anti-*T*. *gondii* IgG may indicate that most women got infected possibly 6 to 12 months before pregnancy, as reported by previous authors [41]. Dating of infection is done with the IgG avidity test, which unfortunately, was a limitation in this study. Generally, women who get infected with toxoplasmosis before pregnancy usually do not transmit the infection to their foetuses because it is believed they have gained immunity. But when infection occurs during pregnancy, the risk of transmission is important. On the other hand, the presence of anti-*T*. *gondii* IgM and cooccurrence of anti-*T*. *gondii* IgM and IgG are a cause for concern and presents a risk for foetal infection. Hence, follow-up test including IgG avidity test are further recommended [42].

Age was found to be positively associated with seropositivity of specific *Toxoplasma* IgG in this study. This is similar to findings in Tanzania [20] and in Nigeria [43]. Younger women, 15-24 years, were more likely infected than their older counter parts, and this is contrary to results obtained in North West Ethiopia [44]. However, in line with a study in Zambia pregnant women 15-24 years old had the highest odds of being infected with *T. gondii* compared to their older counterparts [19]. Other studies in Ethiopia did not find any significant association between age and seroprevalence of *T. gondii* [31].. Educational status of the women was significantly associated to *T. gondii* seroprevalence. Findings from the study revealed that all pregnant women who had no formal level of education were all seropositive for *Toxoplasma* IgG. Furthermore, women with primary level of education were 3.9 times more likely to be infected with *T. gondii* compared to those with secondary level of education. This probably indicates that low level of education is a risk factor for toxoplasmosis among pregnant women. A similar outcome was observed in a study by Nguefack and colleagues where the lower the educational level of subjects was, the higher was the seroprevalence of *T*. *gondii* [22]. Contrary to this, a study in Burkina Faso showed that higher level of education (at least secondary level) was significantly associated with higher odds of being infected compared to those with no formal or primary level of education [45].

Observations from the study revealed that women who had a history of stillbirth had a significantly reduced risk of being seropositive for *Toxoplasma* IgG. This is quite unexpected and contrary to most findings; Li and colleagues in a meta-analysis reported that women with history of stillbirth were more infected with *T. gondii* infection than normal women [9]; Singh and collaborators in India showed that stillbirths were more common in *T. gondii*-infected women than negative ones [46]. On the other hand, no significant associations were found between marital, professional, and HIV statuses, history of abortion, gravidity and parity, water source, eating grilled meat, tasting undercooked meat, presence of cats at home or in the neighbourhood, and *Toxoplasma* antibody seropositivity in the current study. However, some factors were found to be statistically significant in other similar studies: presence of cats at home and history of abortion in Ethiopia [47], HIV status in West Cameroon [26], and source of drinking water in Douala, Cameroon [23].

This is probably the first study that explored neonatal toxoplasmosis from cord blood in Cameroon. Although screening of pregnant women for toxoplasmosis is recommended in hospital settings in Cameroon, it is not obligatory nor a free of cost prenatal screening programme like in some European countries such as France and Austria [48]. Since many newborns do not exhibit clinical signs at birth, performing test only in those with clinical symptoms will likely fail to identify majority of infected infants at birth [1]. Thus, screening of newborn at birth can help to detect infections that were missed during pregnancy thereby detecting any case of congenital infection.

The total toxoplasmosis seroprevalence of 88% obtained from CB in this study was higher than 39.5% obtained in a similar study conducted in Ghana by Kwofie and colleagues [49]. In the present study, the CB seropositivity of IgG only of 55.2% was higher than 39.5% and 19.6% obtained in Ghana [49] and Iran [50], respectively. The presence of specific IgG in CB reflects the presence of specific IgG in maternal serum implying that the mother has been chronically infected or exposed in the past or underwent seroconversion during pregnancy. The seropositivity of IgM anti-*T. gondii*-specific antibody, 7.3% among pregnant women in the present study was quite higher in relation to only one borderline IgM anti-*T*. *gondii* obtained by Nahvi and colleagues [50]. Unlike IgG, IgM antibodies are not transferable via the placental barrier, as such the detection of anti-*T*. *gondii* IgM in CB indicates foetal production of specific IgM and therefore a high likelihood of foetal infection [51]. However, the use of molecular test for confirmation will be reassuring. Furthermore, a strange but intriguing result was observed in this study where 13.1% newborn CB seropositive for IgM were obtained from IgG-negative and IgM-negative pregnant women. Such a scenario may either indicate a false positive result [52] or a case of high mother to child transmission at the end of pregnancy, usually >70% rate. A similar scenario was reported by Sensini in 2006, where a severe case of neonatal toxoplasmosis was demonstrated by positive IgM, IgG, and PCR in a neonate born from a mother seronegative for both IgG and IgM *T*. *gondii* antibodies in repeated serum samples [42]. Worthy of note, majority of samples in this study were collected at the third trimester near birth.

### 4.1. Limitations

Even though the study had as limitation the nonconfirmation of the specific *Toxoplasma* IgM in pregnant women and cord blood by confirmatory tests (IgG avidity and/or PCR test), this finding remains invaluable in signalling the occurrence of infection in pregnant women and their newborn. Secondly, the fact that the assessment of potential toxoplasma risk factors was done retrospectively, it is likely that some element of recall bias may have been introduced.

## 5. Conclusions

Gestational and neonatal toxoplasmosis appears to be more common than earlier reported and the risk of infection higher in younger pregnant women. This highlights extend of the burden of latent *T. gondii* infection in pregnant women and provides knowledge for public health personnel to plan appropriate intervention to mitigate mother to child transmission of toxoplasmosis in Cameroon. However, a countrywide determination of the prevalence of *T*. *gondii* infection in both pregnant women and newborns is invaluable to assert some of the observations. Furthermore, the implementation of obligatory routine low-cost screening algorithm in addition to sensitization of women against various risk factors of toxoplasmosis should be considered an effective way to track and follow up women at risk of transmitting infection to their newborns.

## Figures and Tables

**Table 1:** Sociodemographic, obstetric, clinical, and exposure factors.

Characteristics	Category	Frequency (*n*)	Percentage (%)
Age group (years)	15-24	88	29.3
	25-34	165	55
	35-44	47	15.7

Education	No formal education	3	1.0
	Primary	20	6.7
	Secondary	216	72
	Tertiary	61	20.3

Marital status	Single	147	49
	Married	81	27
	Concubine	72	24

Household income (FCFA)	<50,000	80	26.7
	50,000-100,000	186	62
	>100,000	34	11.3

Profession	Civil servant	15	5.0
	Private sector	47	15.7
	Self-employed	80	26.7
	Student	74	24.7
	Unemployed	84	28.0

HIV status	Positive	27	9.0
	Negative	257	85.7
	Unknown	16	5.3

ANC visits	Less than 4 visits	94	31.3
	4 or more visits	206	68.7

Gravidity	Primigravida (0-1)	79	26.3
	Multigravida [2–4]	164	54.7
	Grandmultigravida (>4)	57	19

Parity	Primipara	134	44.7
	Multipara	166	55.3

History of stillbirth	Yes	15	5
	No	285	95

History of abortion	Yes	97	32.5
	No	203	67.5

Consuming bushmeat	Yes	176	58.7
	No	124	41.3

Habit of eating “soya”	Yes	264	88
	No	36	12

Always disinfecting vegetables/and fruits	Yes	257	85.7
	No	43	14.3

Drinking water source	Treated	271	90.3
	Untreated/mixed	29	9.7

Farm work	Yes	59	19.7
	No	149	80.3

Presence of cats at home	Yes	66	22
	No	234	78

Presence of cats in neighbourhood	Yes	86	33.2
	No	173	66.8

**Table 2:** Seropositivity of *T. gondii* antibodies by sociodemographic, clinical/obstetric history, and exposure habits.

Characteristics	Category	*N*	Prevalence of toxoplasma IgG antibody, *n* (%)	*χ* ^2^ *P* value	Prevalence of toxoplasma IgM antibody, *N* (%)	*χ* ^2^ *P* value
Age group (years)	15-24	88	62 (70.5)	5.962	6 (6.8)	0.131
	25-34	165	133 (80.6)		12 (7.3)	
	35-44	47	41 (87.2)	0.051	4 (8.5)	0.937

Education	No formal education	3	3 (100)	6.996	0 (0)	2.536
	Primary	20	13 (65)	0.072	2 (10)	0.469
	Secondary	216	166 (76.9)		13 (6)	
	Tertiary	61	54 (88.5)		7 (11.5)	

Marital status	Single	147	118 (80.3)	0.486	13 (8.8%)	0.989
	Married	81	63 (77.8)		5 (6.2)	
	Concubine	72	55 (76.4)	0.784	4 (5.6)	0.610

Profession	Civil servant	15	13 (86.7)	5.408	3 (20)	4.047
	Private sector	47	41 (87.2)		4 (8.5)	
	Self-employed	80	61 (76.5)	0.368	5 (6.3)	0.400
	Student	74	59 (72.8)		5 (6.8)	
	Unemployed	81	3 (100)		5 (6)	

Household income	<50,000 FCFA	80	58 (72.5)	2.526	3 (3.8)	2.063
	50,000-100,000 FCFA	186	151 (81.5)		16 (8.6)	
	>100,000 FCFA	34	27 (79.4)	0.283	3 (8.8)	0.356

HIV status	Positive	27	20 (74.1)	1.084	1 (3.7)	2.055
	Negative	257	202 (78.6)		16 (8.6)	
	Unknown	16	14 (87.5)	0.582	3 (8.8)	
Gravidity	Primigravida	79	64 (81)	2.216	4 (5.1)	
	Multigravida	164	124 (75.6)	0.330	14 (8.5)	0.620
	Grandmultivida	57	48 (82.2)		4 (7)	

Parity	Primipa	134	104 (77.6)	2.539	8 (6)	0.763
	Multipa	157	123 (78.3)		13 (8.3)	
	Grandmultipa	9	9 (100)	0.281	1 (11.1)	0.683

ANC visits	Less than 4 visits	94	74 (78.7)	0.001	8 (8.5)	0.003
	More than 4 visits	206	162 (78.6)	0.987	15 (7.4)	0.957

History of stillbirth	Yes	15	9 (60)	3.278	1 (6.7)	0.010
	No	285	227 (79.6)	0.070	21 (7.4)	0.919

History of abortion	Yes	97	79 (81.4)	0.659	7 (7.2)	0.003
	No	203	157 (77.3)	0.417	15 (7.4)	0.957

Consuming bushmeat	Yes	176	137 (77.8)	0.173	8 (8.5)	0.166
	No	124	99 (79.8)	0.677	14 (6.8)	0.683

Habit of tasting undercooked meat	Yes	188	149 (79.3)	0.104	12 (6.4)	0.669
	No	112	87 (72.2)	0.747	10 (8.9)	0.413

Habit of eating “soya”	Yes	264	210 (79.5)	1.012	17 (6.4)	2.587
	No	36	26 (72.2)	0.382	5 (13.9)	0.108

Always disinfecting vegetables/fruits before eating	Yes	257	200 (77.8)	0.764	18 (7)	0.286
	No	43	36 (83.7)	0.382	4 (9.3)	0.593

Drinking water source	Yes	271	212 (78.2)	0.320	20 (7.4)	0.009
	No	29	24 (82.8)	0.883	2 (6.9)	0.924

Presence of cats at home	Yes	66	56 (84.8)	1.927	5 (7.6)	0.007
	No	234	180 (76.9)	0.111	17 (7.3)	0.932

Presence of cats and/other animals in neighbourhood	Yes	95	80 (84.2)	2.546	5 (5.3)	0.877
	No	205	156 (76.1)	0.111	17 (8.3)	0.349

**Table 3:** Risk factors of *Toxoplasma gondii* seroprevalence (*Toxoplasma* IgG antibody) among pregnant women in Yaoundé, Cameroon.

Factors	Category	Crude odd ratio (COR) 95% CI	*P* value	Adjusted odd ratio (AOR) 95% CI	*P* value
Age (years)	15-24	2.866 (1.085-7.570)	0.034	4.649 (1.430-15.112)	0.011
	25-34	1.644 (0.642-4.207)	0.300	2.597 (0.899-7.503)	0.078
	35-44	—	—	—	—

Educational status	No formal education	000	0.999	000	0.999
	Primary	4.154 (1.239-13.930)	0.021	3.940 (1.048-14.817)	0.042
	Secondary	2.324 (0.995-5.428)	0.051	2.234 (0.907-5.500)	0.080
	Tertiary	—	—	—	—

Household income	<50,000FCFA	1.463 (0.557-3.842)	0.440	0.769 (0.259-2.285)	0.636
	50,000-100,000FCFA	0.894 (0.360-2.219)	0.809	0.549 (0.202-1.490)	0.239
	>100,000FCFA	—	—	—	—

Parity	Primipara (0-1)	1.120 (0.643-1.949)	0.689	0.758 (0.391-1.468)	0.411
	Multipara [2–4]	—	—	—	—

History of stillbirth	Yes	0.383 (0.131-1.120)	0.080	0.206 (0.054-0.778)	**0.020**
	No	—	—	—	—

Presence of cats at home	Yes	0.950 (0.477-1.891)	0.883	1.416 (0.643-3.122)	0.388
	No	—	—	—	—

Presence of cats and/or other animals in the neighbourhood	Yes	1.675 (0.885-3.171)	0.113	1.481 (0.751-2.919)	0.257
	No	—	—	—	—

## Data Availability

The data sets analysed during the current study are available from the corresponding author on reasonable request.
